# Proteomic insights into molecular alterations associated with Kawasaki disease in children

**DOI:** 10.1186/s13052-025-01853-8

**Published:** 2025-02-21

**Authors:** Chengyi Wang, Wenxin Yu, Xinyue Wu, Shibiao Wang, Lumin Chen, Guanghua Liu

**Affiliations:** 1 College of Clinical Medicine for Obstetrics & Gynecology and Pediatrics, Fujian Medical University, No. 966 HengYu Road, Jinan District, Fuzhou, Fujian 350001 PR China; 2 Department of Pediatrics, Fujian Children’s Hospital (Fujian Branch of Shanghai Children’s Medical Center), College of Clinical Medicine for Obstetrics & Gynecology and Pediatrics, Fujian Medical University, Fuzhou, 350001 PR China; 3 Pediatric Intensive Care Unit, Fujian Children’s Hospital (Fujian Branch of Shanghai Children’s Medical Center), College of Clinical Medicine for Obstetrics & Gynecology and Pediatrics, Fujian Medical University, Fuzhou, 350001 PR China; 4 Fujian Children’s Hospital (Fujian Branch of Shanghai Children’s Medical Center), College of Clinical Medicine for Obstetrics & Gynecology and Pediatrics, Fujian Medical University, No. 966 HengYu Road, Jinan District, Fuzhou, Fujian 350001 PR China

**Keywords:** Complement component 6, Complement component 3, α1-antitrypsin, Proteomic, Kawasaki disease

## Abstract

**Background:**

Kawasaki disease (KD) is a pediatric vasculitis that can lead to coronary artery complications if not promptly diagnosed. Its nonspecific early symptoms, primarily fever, often result in misdiagnosis. This study aimed to identify potential biomarkers for early KD diagnosis using proteomic analysis of blood samples.

**Methods:**

Serum samples were collected from three groups: children with acute KD (*n* = 20, CQB group), age-matched febrile children with bacterial infections (*n* = 20, C group), and children recovered from KD (*n* = 8, CQBC group). Proteomic analysis was performed to identify differentially expressed proteins in serum specimens, followed by functional and pathway enrichment analysis.

**Results:**

Compared to controls, 92 proteins were upregulated and 101 were downregulated in acute KD, with significant enrichment in the AMPK pathway. In recovered KD, 537 proteins were upregulated and 231 downregulated, predominantly affecting the PI3K-Akt pathway. A total of 56 proteins showed contrasting expression patterns between acute and recovery phases, implicating the complement and coagulation cascades. Notably, complement component 6 (C6), complement component 3 (C3), and α1-antitrypsin (A1AT) emerged as potential biomarkers involved in KD progression and recovery.

**Conclusions:**

C6, C3, and A1AT may serve as novel biomarkers for early KD diagnosis and monitoring. These findings provide new insights into KD pathogenesis and potential targets for clinical application.

## Introduction

Kawasaki disease (KD) is a common systemic vasculitis primarily affecting children under five years of age [1]. It is a leading cause of acquired heart disease in this population. If not diagnosed and treated promptly, KD can lead to serious complications such as coronary artery lesions (CAL), aneurysms, and long-term cardiovascular problems [2]. The pathophysiology of KD involves a multifaceted and poorly understood immune response, characterized by systemic vascular inflammation, endothelial dysfunction, and immune cell infiltration [3]. Platelet activation and thrombocytosis, which are hallmark features of KD, exacerbate vascular injury and increase the risk of coronary artery damage [4]. Despite extensive research, the exact molecular mechanisms that drive KD are still unclear, complicating both its diagnosis and treatment.

In recent years, there has been growing interest in identifying biomarkers for KD that can facilitate early diagnosis and improve prognosis [5]. Biomarkers can help distinguish KD from other febrile illnesses and provide valuable insights into disease activity and response to treatment [6, 7]. This is especially critical for distinguishing incomplete KD, where clinical manifestations are less apparent, leading to delays in diagnosis [8, 9]. Proteomics has emerged as a powerful tool for discovering disease-specific biomarkers, offering a more comprehensive approach than transcriptomics. While transcriptomics provides data at the gene expression level, proteomics captures the functional changes in proteins, including post-translational modifications, which are crucial for understanding cellular processes and disease mechanisms [10–12]. These functional changes at the protein level provide a more direct correlation to disease activity, aiding clinicians in interpreting complex disease patterns. This approach is particularly relevant for KD, where the interplay of immune, vascular, and metabolic pathways is complex and cannot be fully elucidated through mRNA-level analyses [13].

Proteomic approaches have the potential to offer deeper insights into the pathophysiological mechanisms of KD. For example, pathways such as AMPK and PI3K-Akt, which are involved in vascular inflammation, endothelial repair, and cellular survival, have been implicated in KD [14, 15]. Furthermore, complement and coagulation cascades have been highlighted as key players in both the acute and resolution phases of KD, indicating their dual roles in immune defense and vascular repair [16]. These findings underscore the need for targeted research to validate these pathways’ roles in KD and their potential for clinical application. These pathways not only provide a window into KD pathophysiology but also serve as potential therapeutic targets.

Despite its potential, proteomics research in KD has yet to reach its full translational potential. This study bridges this gap by utilizing proteomic technologies to analyze serum samples from children with KD, identify differentially expressed proteins, and uncover the molecular mechanisms underlying the disease. Through functional and pathway enrichment analyses, we seek to identify novel biomarkers and therapeutic targets that could aid in the early diagnosis and management of KD.

## Materials and methods

### Subjects and study design

Children with KD hospitalized at our tertiary children’s hospital in Fujian Province, China (*n* = 20) between January 2018 and December 2020 were included in the CQB group. Age-matched febrile children (*n* = 20) admitted during the same period due to bacterial infections formed the infection control group (C group), while children who had recovered from KD (*n* = 8) were classified as the CQBC group.

KD diagnoses were made according to the 2017 Guidelines of the American Heart Association (AHA). Exclusion criteria included cases beyond the acute phase of KD, a disease duration exceeding 10 days, congenital heart defects, prior treatment, or incomplete medical records. Medical records of included participants were reviewed, encompassing age, gender, clinical manifestations, blood routine indices, and serum biochemical indices.

This study was approved by the Ethics Committee of Fujian Maternity and Child Health Hospital (No. 199 [2018]), and informed consent was obtained from all participants’ families. All procedures adhered to institutional and national ethical standards and the Helsinki Declaration (1964) and its later amendments.

### Proteomics analysis

#### Sample preparation and fractionation to generate DDA library

Fasting venous blood samples from children with KD were collected for proteomic analysis within 48 h before and after intravenous immune globulin (IVIG) treatment. Similarly, fasting venous blood samples from the infection control group were collected within 48 h of admission for proteomic analysis. After centrifugation at 12,000×g for 10 min at 4 °C, the clear supernatants were carefully separated and stored at -80 °C. Agilent technology was employed to remove the most prevalent proteins from plasma samples, leveraging human 14/mouse 3 multiple affinity reagents [17]. Following this, proteins of high and low abundance were isolated and collected individually. The desalination and concentration of these fractions were achieved using ultrafiltration membranes with a 5 kDa molecular weight cutoff. Subsequently, the samples underwent treatment with an SDT buffer solution, consisting of 4% SDS, 100 mM DTT, and 150 mM Tris-HCl at pH 8.0, and were then heated to boiling point for 15 min. After centrifugation at 14,000 g for 20 min, the protein concentration in the resulting supernatant was measured using the BCA Protein Assay Kit. The samples were stored at -80 °C for long-term preservation.

#### Filtration assisted sample preparation digestion procedure

A protein sample of 200 µg was processed through an ultrafiltration device (using a Microcon unit with a 10 kD cutoff) [18]. It was washed with an ultrafiltration buffer (UA buffer, containing 8 M urea and 150 mM Tris-HCl at pH 8.0) to remove detergent, DTT, and other substances. Subsequently, 100 µl of iodoacetamide was added to alkylate the cysteine residues, followed by a 30-minute incubation in the dark. The protein sample was triple-washed with 100 µl of UA buffer and rinsed twice with 100 µl of 25 mM NH4HCO3 buffer. Trypsin (4 µg) in 40 µl of 25 mM NH4HCO3 buffer was added for digestion.

The digested peptides were desalted and gathered after vacuum centrifugation in 40 µl of a 0.1% (v/v) formic acid solution. The peptide concentration was determined using UV spectroscopy at 280 nm. Peptides were fractionated into 10 distinct groups using a high pH reversed-phase peptide fractionation kit from Thermo Scientific™ Pierce™, concentrated using a vacuum centrifuge, and recombined in 15 µl of 0.1% (v/v) formic acid solution. Desalting was performed using Empore™ SPE C18 columns (7 mm inner diameter, 3 ml volume), followed by reconstitution in 40 µl of 0.1% (v/v) formic acid solution [19]. iRT standards from Biognosys were incorporated to calibrate retention time, with a set ratio of 1:3 between iRT and sample peptides.

#### DDA mass spectrometry

The DDA library-derived fractions were analyzed using a Thermo Fisher Scientific Q Exactive HF-X mass spectrometer interfaced with an Easy nLC 1200 chromatography system [20]. A 1.5 µg peptide sample was applied to an EASY-Spray™ C18 trap column (Thermo Scientific, P/N 164946, 3 μm, 75 μm × 2 cm) before separation on an EASY-Spray™ C18 LC analysis column (Thermo Scientific, ES802, 2 μm, 75 μm × 25 cm). The peptides were eluted at a flow rate of 250 nl/min over a 120-minute gradient using buffer B (84% acetonitrile, 0.1% formic acid).

The mass spectrometer operated in a scanning range of 300–1800 m/z, with a resolution of 60,000 at 200 m/z. It utilized target AGC settings of 3e6, a maximum ion time of 25 ms, a dynamic exclusion duration of 30.0 s, and a normalized collision energy setting of 30 eV. Each MS-SIM scan was followed by 20 ddMS2 scans.

#### Mass spectrometry analysis is used for data-independent acquisition (DIA)

Peptides from each sample were examined using liquid chromatography-tandem mass spectrometry (LC-MS/MS) in DIA mode [21]. Each DIA cycle comprised a full MS-SIM scan accompanied by 30 DIA scans spanning the m/z range of 350–1800. The parameters were configured as follows: the SIM full scan resolution was set at 120,000 at 200 m/z; the automatic gain control (AGC) was set to 3e6; and the maximum ion time (IT) was 50 ms. For the DIA scans in profile mode, the resolution was 15,000; the AGC target was 3e6; the maximum IT was set to automatic; and the normalized collision energy was maintained at 30 eV. A linear gradient of buffer B, consisting of 84% acetonitrile and 0.1% formic acid, was applied at a flow rate of 250 nl/min over a period of 120 min. Quality control (QC) samples were introduced into the DIA mode at the commencement of the mass spectrometry analysis and following every sixth injection during the course of the experiment to ensure consistent MS performance.

#### Mass spectrometry data analysis

The Specronaut™ software version 14.4.200727.47784 was utilized to interrogate the FASTA Sequence Database. The parameters were configured as follows: the enzyme specificity was set to trypsin, with a maximum of two missed cleavages allowed; the fixed modification was set to carbamoylation at cysteine (C); and the dynamic modifications included oxidation at methionine (M) and acetylation at the protein N-terminus. Protein identifications were ascertained with a confidence level of 99%, and the false discovery rate (FDR) was calculated using the formula FDR = N (bait) * 2 / (N (bait) + N (target)) to ensure it was less than or equal to 1%. The spectral library was constructed by integrating the raw data file and DDA search results into Specronaut Pulsar X TM_12.0.20491.4 from Biognosys. The key software parameters were set as follows: dynamic iRT was selected for retention time prediction; MS2 level interference correction and cross-run normalization were activated. All results were filtered to ensure an FDR of ≤ 1%.

### Bioinformatics analysis

GO analysis of differentially expressed proteins was conducted, including biological processes (BP), cellular components (CC), and molecular functions (MF) [22]. Protein-protein interactions (PPIs) were mapped using the STRING database (version 10.0) [23]. Subcellular localization predictions were made with WolfPsort software (version 0.2) [24]. InterProScan was used for domain predictions, and domain enrichment analysis was performed using Fisher’s Exact Test [25]. KEGG pathway enrichment analysis was conducted for clustering molecular interactions, reactions, and networks [26]. Data visualizations were created using the R package ggplot2 [27].

### Statistical analysis

Statistical analysis was performed using IBM SPSS, version 23.0 (Chicago, USA). Descriptive analyses were conducted, with results expressed as mean ± standard deviation (SD) for normally distributed variables. Variance between the C group and CQB group, as well as between the CQB group and CQBC group, was assessed using Student’s t-test for normally distributed variables. A P-value of < 0.05 was considered statistically significant.

## Results

### Clinical features of the study population

Children diagnosed with KD before IVIG treatment and hospitalized in our hospital (*n* = 20) were included in the CQB group. Febrile children (*n* = 20) admitted to our hospital for treatment due to bacterial infection were included in the infection control (C) group. Children with KD after IVIG treatment (*n* = 8) were classified as the CQBC group. The study was approved by the ethics committee of the University (No. 2018 − 199). Informed consent was obtained from all patients and their families, and patient data were analyzed anonymously.

Significant differences were observed among the three groups (Table 1) in the rates of conjunctival hyperemia, skin rashes, fissured lips, lymph node enlargement, changes in extremities, as well as the average levels of platelet count, erythrocyte sedimentation rate, serum sodium, serum chloride, and alanine transaminase. Other factors, such as gender and age, showed no statistical significance.

**Table 1:** Comparison of characteristics between KD before and after IVIG treatment and febrile patients with bacterial infection

Characteristics	All (*n* = 48)	KD before IVIG treatment (CQB Group *n* = 20)	KD after IVIG treatment (CQBC Group *n* = 8)	febrile Patients with bacterial infection( C Group *n* = 20)	F/H/ X^2^	*P* - Value
Age (m)	25.22 ± 22.91	23.35 ± 15.29	24.23 ± 10.32	27.50 ± 31.87	0.167	0.846
Gender (n,% males)	35-72.9	15–75	5-62.5	15–75	0.527	0.768
Maximum temperature > 39℃ (n,%)	31-64.6	13–65	6–75	12–60	0.565	0.754
Fever > 7d (n,%)	11-22.9	4–20	3-37.5	4–20	1.156	0.561
Fever > 10d (n,%)	3-6.3	0–0	0–0	3–15	4.480	0.106
Conjunctival hyperemia (n,%)	26-54.2	18–90	8-100	0–0	40.750	< 0.001
Skin mashes (n,%)	31-64.6	18–90	8-100	5–25	23.736	< 0.001
Fissured lips (n,%)	28-58.3	20–100	8-100	0–0	48.000	< 0.001
Enlargement of lymph nodes (n,%)	32-66.7	18–90	8-100	6–30	21.000	< 0.001
Changes in extremities (n,%)	21-43.8	15–75	6–75	0–0	26.667	< 0.001
White blood cell count x 10^9^/L	13.72 ± 4.88	14.16 ± 4.68	16.20 ± 3.39	12.29 ± 5.27	2.064	0.139
Neutrophil percentage (%)	61.10 ± 17.13	62.61 ± 16.74	68.20 ± 16.76	56.75 ± 17.29	1.436	0.249
Neutrophil counts, x 10^9^/L	8.57 ± 4.08	8.80 ± 3.82	11.04 ± 2.97	7.35 ± 4.37	2.540	0.090
Neutrophil counts /Lymphocyte counts	3.31 ± 2.99	3.55 ± 2.98	5.04 ± 4.57	2.37 ± 1.82	2.562	0.088
Red blood cell volume distribution width (%)	37.91 ± 4.81	37.77 ± 6.46	36.78 ± 2.94	38.51 ± 3.38	0.374	0.690
Hemoglobin, g/L	115.65 ± 11.76	113.15 ± 12.53	114.63 ± 13.08	118.55 ± 10.30	1.095	0.343
Platelet count, x 10^9^/L	564.98 ± 208.34	633.10 ± 255.24	616.50 ± 120.01	476.25 ± 150.44	3.454	0.040
C-reactive protein, mg/L	66.89 ± 49.89	81.46 ± 39.31	67.21 ± 48.37	52.19 ± 57.44	1.777	0.181
erythrocyte sedimentation rate	65.96 ± 36.22	81.40 ± 29.84	87.75 ± 16.32	41.80 ± 34.50	10.999	< 0.001
Serum sodium, mmol/L	135.30 ± 3.27	134.01 ± 2.86	135.36 ± 1.30	136.57 ± 3.76	3.382	0.043
Serum chloride, mmol/L	103.72 ± 2.78	102.73 ± 2.29	103.00 ± 1.51	105.00 ± 3.18	4.129	0.023
Serum potassium, mmol/L	4.35 ± 0.50	4.30 ± 0.54	4.39 ± 0.47	4.38 ± 0.49	0.153	0.859
Serum calcium, mmol/L	2.35 ± 0.13	2.32 ± 0.13	2.34 ± 0.10	2.39 ± 0.14	1.463	0.242
Alanine transaminase, U/L	23.20(15.85–97.48)	74.70(19.68–140.90)	71.59(16.00-157.80)	17.88(15.48–23.24)	9.221	0.010
Aspartate Aminotransferase, U/L	53.36 ± 38.56	68.53 ± 49.94	48.58 ± 24.56	40.10 ± 22.90	3.035	0.058
Total Bilirubin,µmol/L	8.47 ± 8.25	9.96 ± 11.56	8.76 ± 7.08	6.87 ± 3.32	0.697	0.504
Total Cholesterol, mmol/L	3.74 ± 0.94	3.76 ± 1.00	4.21 ± 1.17	3.54 ± 0.74	1.482	0.238
Triglyceride, mmol/L	2.03 ± 3.28	1.58 ± 1.07	2.50 ± 1.87	2.29 ± 4.87	0.325	0.724
Low-Density Lipoprotein, mmol/L	2.30 ± 0.63	2.45 ± 0.59	2.47 ± 0.66	2.08 ± 0.62	2.178	0.125
High-Density Lipoprotein, mmol/L	0.87 ± 0.28	0.85 ± 0.32	0.68 ± 0.25	0.97 ± 0.21	3.441	0.041
Albumin, g/L	38.24 ± 5.80	35.83 ± 5.89	36.20 ± 6.75	41.47 ± 3.61	6.562	0.003
Lactate dehydrogenase, U/L	356.68 ± 100.57	360.57 ± 88.93	368.73 ± 60.35	347.97 ± 124.96	0.142	0.868
Creatine kinase, U/L	88.14 ± 72.81	108.73 ± 95.83	45.65 ± 33.31	84.55 ± 47.96	2.308	0.111
Creatine Kinase MB, U/L	38.02 ± 28.27	36.42 ± 16.28	35.48 ± 15.67	40.63 ± 40.11	0.144	0.866
Length of hospitalization (d)	7.94 ± 3.24	8.75 ± 3.48	7.87 ± 3.83	7.15 ± 2.66	1.235	0.301

### Identification of differentially abundant proteins in the CQB/C group

The proteomics experimental workflow primarily encompasses procedures such as protein extraction, enzymatic digestion into peptides, chromatographic separation, LC-MS/MS analysis, DDA data collection, and database queries. Post-experiment, a series of bioinformatics analyses were conducted, including protein identification, differential expression analysis, and functional profiling.

**Fig. 1:**
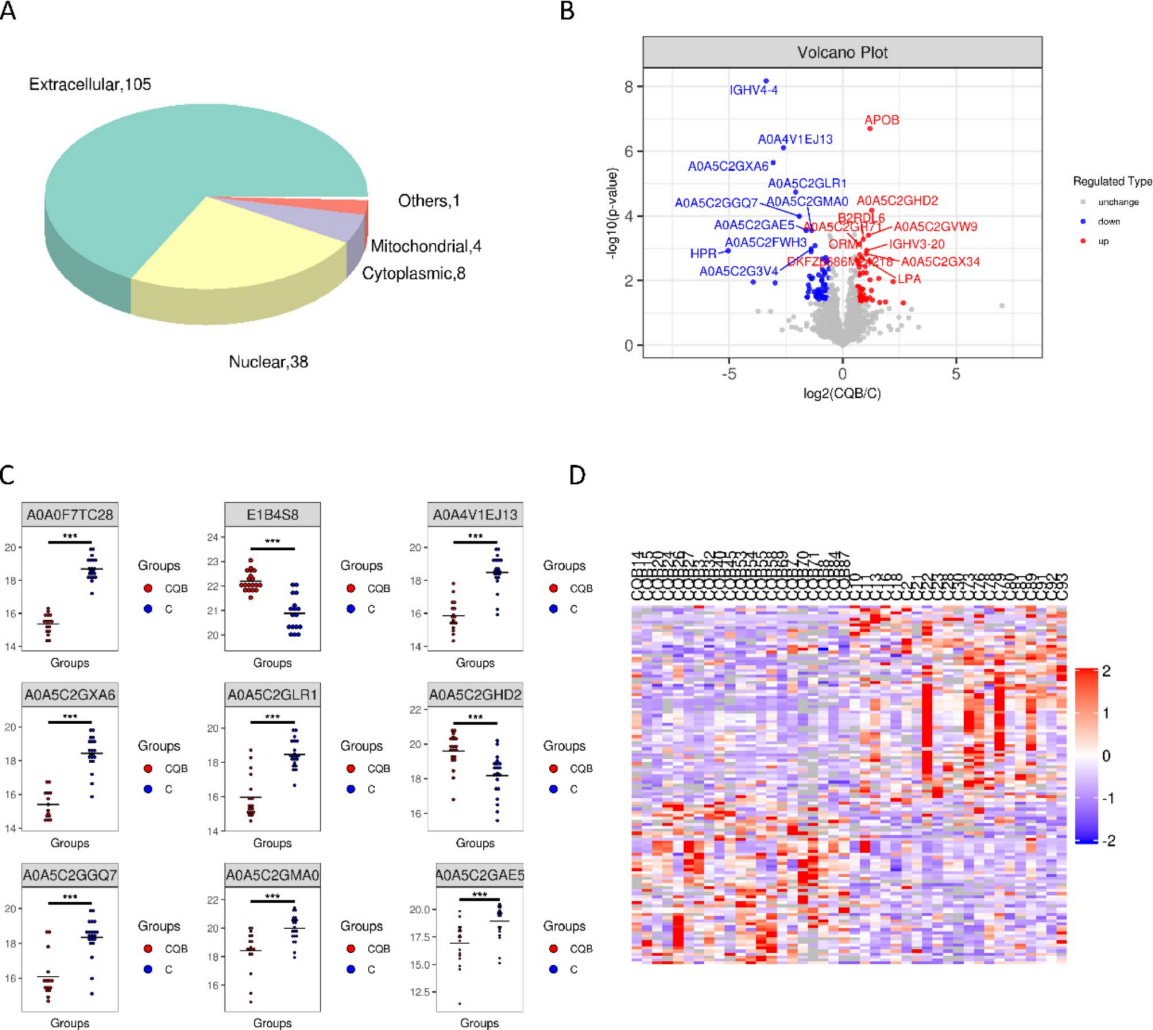
Analysis of differential protein expression within the CQB/C group. (**A**) Pie chart illustrating the distribution of subcellular localizations for proteins with differential expression in the CQB/C group. (**B**) Volcano plot comparing protein expression levels between the C and CQB groups. (**C**) Boxplot with the x-axis representing the groups and the y-axis showing protein expression levels on a base-2 logarithmic scale. Red and blue elements highlight the expression levels of differentially expressed proteins across samples. (**D**) Heatmap showing hierarchical clustering of significantly differentially expressed proteins, with each column representing a sample (x-axis) and each row corresponding to a protein (y-axis), illustrating their expression patterns across different samples

Initially, a subcellular localization study was carried out on the proteins exhibiting differential expression between the CQB and C groups. Figure 1A illustrates that the majority of these proteins (105) were predominantly localized in the extracellular space, followed by 38 in the nucleus, 8 in the cytoplasm, and 4 in the mitochondria.

When screening for differentially abundant proteins, a threshold of fold change (FC) greater than 1.5 (indicating an increase of more than 1.5-fold) or less than 0.67 (indicating a decrease to less than 0.67-fold) with a P-value less than 0.05 was applied. This analysis identified 92 proteins that were up-regulated and 101 that were down-regulated. Figure 1B presents a volcano plot to visualize the significant differences in protein expression. Proteins significantly down-regulated are shown in blue (FC < 0.67 and *P* < 0.05), and those significantly up-regulated are shown in red (FC > 1.5 and *P* < 0.05). Non-differentially expressed proteins are indicated in gray. The top 10 most significantly up-regulated and down-regulated proteins are specifically marked.

To further connect these findings to clinical relevance, the identified proteins were analyzed for their potential as biomarkers. For instance, complement component 3 (C3), which was significantly up-regulated, is known to play a critical role in immune modulation, endothelial repair, and KD pathophysiology. This highlights its potential utility in differentiating between acute and recovery phases of KD.

A hierarchical clustering analysis was performed to evaluate expression profiles between and within groups, verifying the project’s group allocation logic and confirming the biological relevance of the identified differential proteins. Figure 1D depicts a heatmap of the clustered differentially expressed proteins, further underscoring potential clinical markers such as α1-Antitrypsin, which has implications for vascular repair.

### Identification of proteins function in CQB/C group

**Fig. 2:**
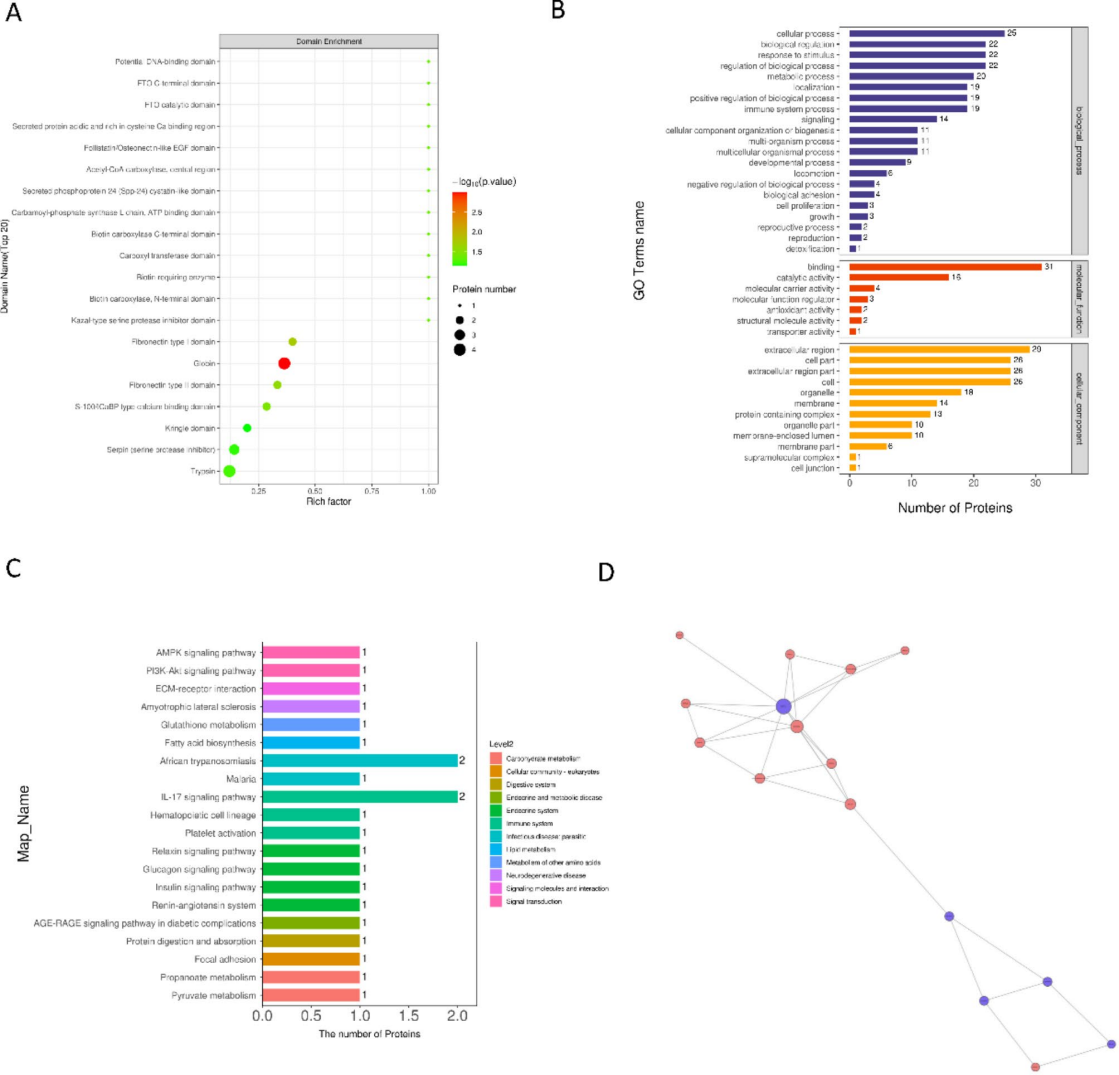
Functional analysis of proteins in the CQB/C group. (**A**) The x-axis represents the enrichment factor, with a threshold of Rich Factor ≤ 1. The y-axis shows the statistical outcomes for differential proteins within each domain category; bubble coloration indicates the level of significance for each enriched domain category. (**B**) The y-axis in the figure denotes GO level 2 details encompassing BP, MF, and CC, color-coded as blue, red, and orange, respectively; the x-axis (bottom) signifies the quantity of differentially expressed proteins classified under each functional group. (**C**) Histogram displaying KEGG pathway assignments and attributes for differentially expressed proteins when comparing the CQB and C groups. (**D**) Interaction network diagram of differentially expressed proteins between the CQB and C groups

Domains associated with differentially expressed proteins were predicted, revealing that these proteins predominantly belong to domains such as globin, trypsin, serpin (serine protease inhibitors), and the Kringle domain (Fig. 2A). GO annotation provided insights into the proteins’ roles, cellular locations, and involvement in biological pathways. Differentially expressed proteins were primarily enriched in BP categories such as cellular processes, biological regulation, response to stimuli, and regulation of biological processes. In MF, they were associated with binding, catalytic activity, and molecular carrier activity, while in CC, they were localized to the extracellular region, cell parts, and extracellular region parts (Fig. 2B).

KEGG pathway enrichment analysis revealed that pathways like the AMPK signaling pathway were significantly affected in the CQB group (Fig. 2C). The dysregulation of this pathway is relevant to KD as it influences endothelial function, inflammation resolution, and vascular repair. For example, AMPK activation has been implicated in reducing inflammation and promoting endothelial repair, highlighting its potential as a therapeutic target. A PPI network diagram, constructed using STRING database data, represents the differential expression patterns of proteins within this group (Fig. 2D).

### Identification of differentially abundant proteins in CQBC/CQB group

**Fig. 3:**
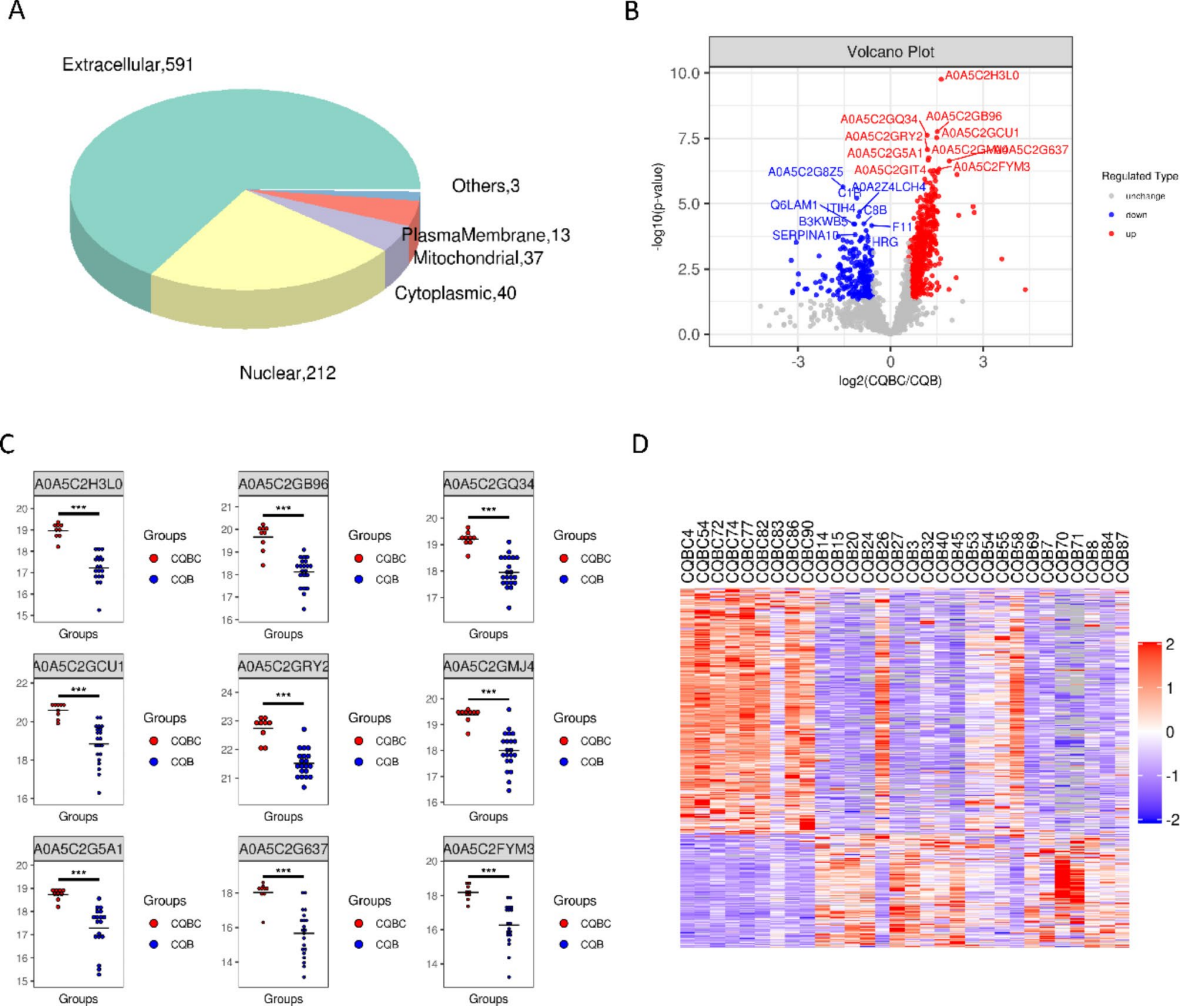
Analysis of differential protein expression within the CQBC/CQB group. (**A**) Pie chart illustrating the distribution of subcellular localization for proteins with differential expression in the CQBC/CQB group. (**B**) Volcano plot comparing the CQB group to the CQBC group. (**C**) Boxplot where the x-axis indicates Group, the y-axis represents protein expression levels following a base-2 logarithmic transformation; red and blue elements in the plot signify the expression levels of differentially expressed proteins across various samples. (**D**) Hierarchical clustering depicted as a dendrogram heatmap, with each column representing a sample set (x-axis displays sample details), each row corresponds to a protein (y-axis lists significantly differentially expressed proteins), showing the expression patterns of significantly differential proteins across different samples

The subcellular localization analysis of differentially expressed proteins within the CQBC/CQB group showed that 591 were predominantly found in the extracellular space, 212 in the nucleus, 40 in the cytoplasm, 37 in the mitochondria, and 13 in the plasma membrane (Fig. 3A). Between the CQBC and CQB groups, 537 proteins were up-regulated and 231 were down-regulated. Figure 3B highlights the top 10 proteins with the most notable expression changes. Proteins such as A0A5C2H3L0, A0A5C2GB96, and A0A5C2GQ34 exhibited significant up-regulation in the CQBC group (Fig. 3C). Hierarchical clustering further illustrated these group differences (Fig. 3D).

### Identification of protein functions in the CQBC/CQB group

**Fig. 4:**
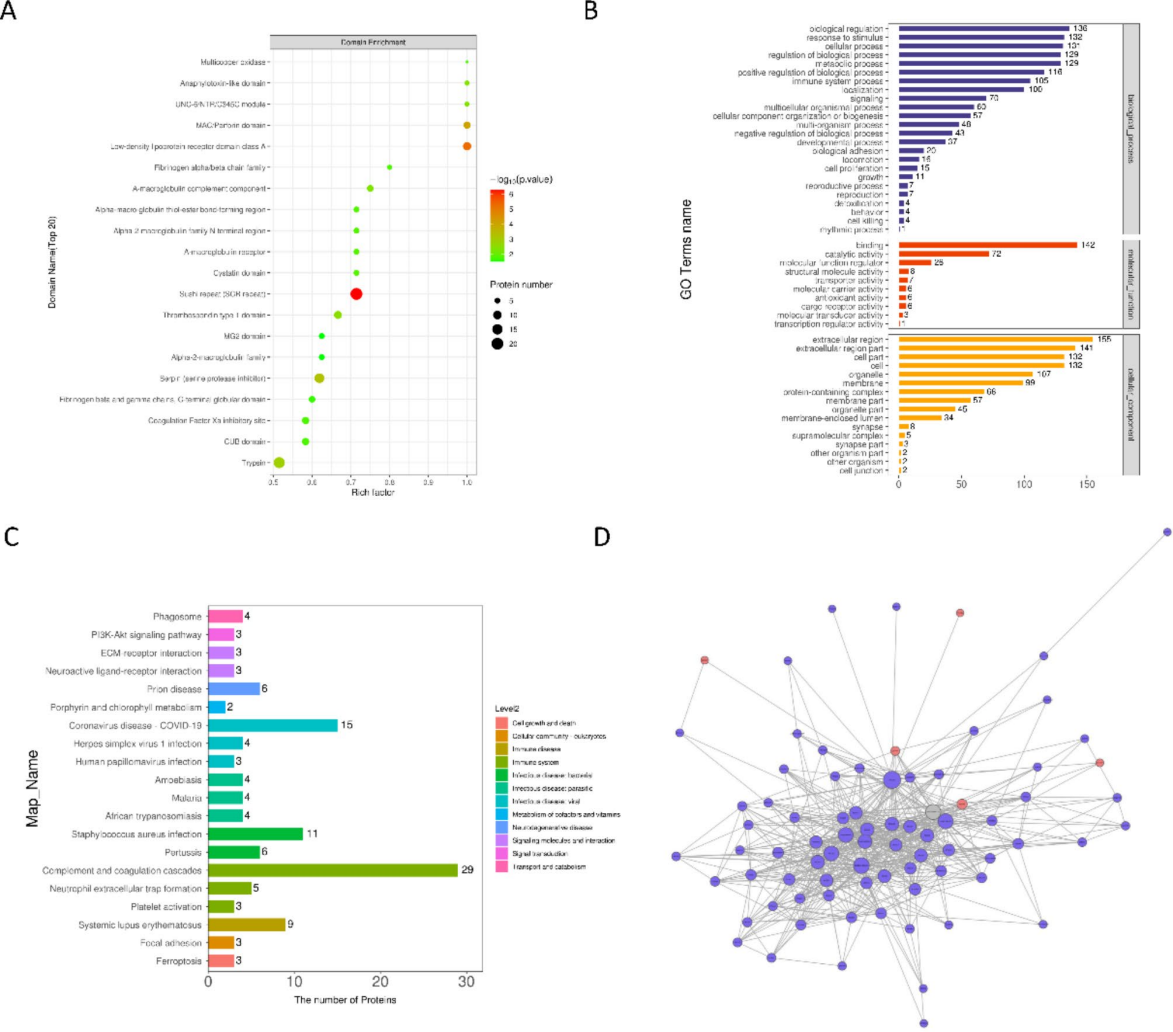
Functional analysis of proteins in the CQBC/CQB group. (**A**) The x-axis represents the enrichment factor, capped at Rich Factor ≤ 1. The y-axis displays the statistical analysis for differential proteins within each domain category, with bubble coloration indicating the significance level of the domain enrichment. (**B**) The y-axis in the figure denotes GO level 2 information, including biological processes, molecular functions, and cellular components, color-coded blue, red, and orange respectively; the x-axis (bottom) shows the count of differentially expressed proteins associated with each functional category. (**C**) Histogram presenting KEGG pathway assignments and characteristics for differentially expressed proteins between the CQB and CQBC groups. (**D**) Interaction network visualization of differentially expressed proteins for the CQB and CQBC groups

Domain analysis of the differentially expressed proteins in the CQBC/CQB group revealed associations with Sushi repeat (SCR repeat), low-density lipoprotein receptor class A domain, and MAC/Perforin domain (Fig. 4A). GO analysis indicated enrichment in BP such as biological regulation, response to stimuli, and cellular processes. In MF, the proteins were associated with binding, catalytic activity, and molecular function regulation, while in CC, they were localized to the extracellular region and cell parts (Fig. 4B). KEGG pathway analysis indicated alterations in the PI3K-Akt signaling pathway, which is critically involved in vascular inflammation and repair. This pathway has been linked to coronary artery lesion (CAL) development in KD and could serve as a potential therapeutic target (Fig. 4C). A PPI network diagram was constructed for these proteins (Fig. 4D). These results underscore the dynamic changes in signaling pathways during the progression and resolution of KD.

### Key proteins involved in KD progression by combining CQB/C Group and CQBC/CQB group

**Fig. 5:**
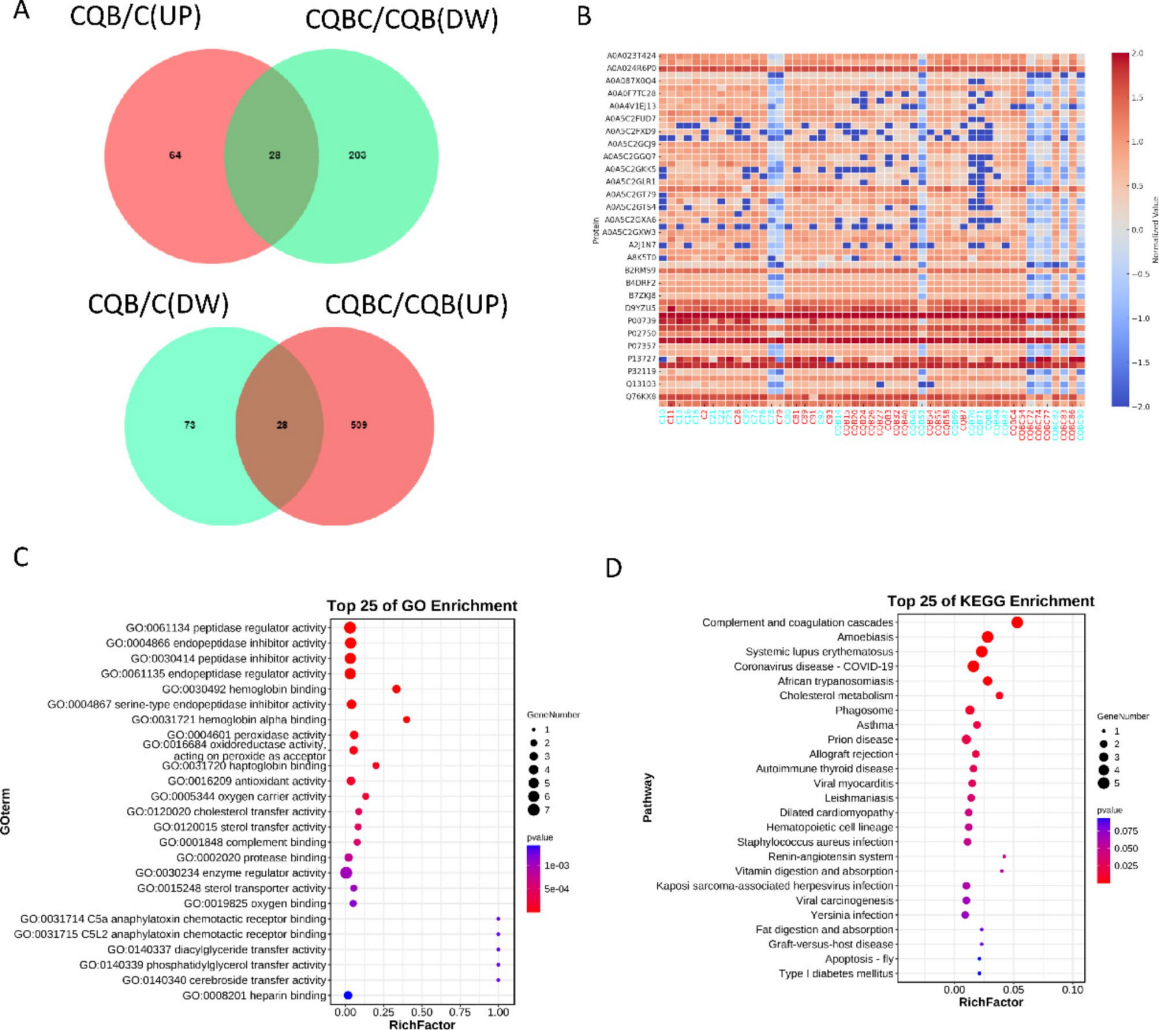
Key proteins involved in KD progression by combining CQB/C group and CQBC/CQB group. (**A**) Venn diagram of proteins meeting the requirements for differential expression. (**B**) Heatmap of proteins meeting the requirements for differential expression. (**C**) GO analysis of the function of proteins. (**D**) Bubble diagram of KEGG enrichment pathway

We identified 56 differentially expressed proteins that exhibited both elevated expressions in the CQB/C group and decreased expressions in the CQBC/CQB group, or vice versa. A Venn diagram illustrates this overlap (Fig. 5A). A heatmap further highlights these proteins (Fig. 5B). GO enrichment analysis revealed that these proteins are involved in activities such as peptidase regulation, endopeptidase inhibition, and peptidase inhibition (Fig. 5C). KEGG pathway analysis identified complement and coagulation cascades as key pathways, with notable contributions from complement component 6 (C6), complement component 3 (C3), and α1-Antitrypsin (Fig. 5D). These findings suggest that these proteins play critical roles in immune modulation and vascular repair, providing potential biomarkers for clinical differentiation between complete and incomplete KD, CAL-positive and CAL-negative cases, and KDSS vs. responsive KD cases.

## Discussion

Kawasaki disease (KD) is a pediatric vasculitis characterized by fever and other nonspecific clinical manifestations, which often leads to delayed diagnosis and misdiagnosis as bacterial infections. This misdiagnosis can result in inappropriate anti-infective therapy and delayed administration of gamma globulin, which is critical for preventing coronary artery lesions (CAL) and other complications. Given the difficulty in diagnosing incomplete KD, the identification of reliable biomarkers is crucial for improving early diagnosis and clinical outcomes for children with unexplained fever [28, 29]. Our study aimed to explore proteomic alterations associated with KD, providing insight into potential biomarkers and therapeutic pathways.

Our proteomic analysis identified 105 differentially expressed proteins in the CQB/C group. These proteins were predominantly localized in the extracellular space, with notable changes observed in proteins such as A0A0F7TC28, A0A4V1EJ13, and others, which were significantly down-regulated in the CQB group. Conversely, proteins like E1B4S8 and A0A5C2GHD2 were markedly up-regulated. The clinical relevance of these findings lies in their potential as biomarkers for distinguishing between subtypes of KD, including incomplete KD, which is often more challenging to diagnose. Functional analysis revealed that the differentially expressed proteins were involved in key biological processes, such as cellular response to stimuli and biological regulation, which are crucial in understanding the inflammatory and vascular responses in KD.

Our study also revealed significant alterations in the AMPK pathway in the CQB group. AMPK regulates cellular energy balance and plays a pivotal role in maintaining cellular and whole-body energy homeostasis [30]. Beyond its metabolic functions, AMPK has been implicated in modulating inflammatory responses, mitigating endothelial dysfunction, and reducing vascular injury, all of which are central to the progression of KD [31, 32]. One of the mechanisms by which activated AMPK exerts its protective effects is through promoting NADPH synthesis, thereby decreasing ROS accumulation and suppressing NF-κB activation [33]. This cascade ultimately leads to reduced TNF-α production, a key inflammatory mediator in KD. TNF-α plays a critical role in local inflammation and coronary artery damage, as it stimulates vascular endothelial cells to express intercellular adhesion molecule-1 (ICAM-1) and monocyte chemoattractant protein-1 (MCP-1), facilitating inflammatory cell infiltration into affected tissues [34]. Additionally, AMPK has been shown to inhibit mTOR via direct phosphorylation of TSC2 and Raptor, further suppressing NF-κB activity and contributing to its anti-inflammatory effects [35]. Evidence from prior studies supports the protective role of AMPK activation in KD, with findings suggesting that it can attenuate inflammation and prevent apoptosis in endothelial cells through modulation of the AMPK/mTOR/NF-κB pathway [36]. Similarly, cordycepin has been demonstrated to reduce TNF-α production via AMPK activation, reinforcing the therapeutic potential of targeting this pathway [37]. These findings underscore the promise of AMPK as a therapeutic target in KD, with potential to ameliorate inflammation and protect against vascular damage. Further research is warranted to elucidate the precise mechanisms by which AMPK influences coronary artery lesions and to evaluate the clinical efficacy of AMPK-targeted therapies in improving outcomes for KD patients.

Additionally, our analysis revealed significant alterations in the PI3K-Akt pathway in the CQBC group. This pathway regulates endothelial cell survival, proliferation, and inflammation, making it highly relevant in the context of KD, where vascular injury and CAL are primary concerns [38]. Studies have shown that modulating the PI3K/Akt axis can protect endothelial cells from inflammatory damage induced by mediators such as TNF-α [39, 40] Given the pivotal role of PI3K/Akt in vascular damage, targeting this pathway could represent a novel therapeutic approach to prevent and manage CAL in KD patients. Berberine, which modulates PI3K/Akt, has demonstrated protective effects in endothelial cells [39], suggesting that similar therapeutic strategies could be effective in the management of KD.

Moreover, combining the CQB/C and CQBC/CQB groups allowed us to identify 56 differentially expressed proteins, which were either up-regulated in the CQB/C group and down-regulated in the CQBC/CQB group, or vice versa. KEGG pathway analysis revealed that the complement and coagulation cascades play a significant role in the development and resolution of KD. Complement components C3 and C6, along with α1-Antitrypsin, were notably involved. The immune-inflammatory response and endothelial dysfunction contribute to CAL in KD [41]. The involvement of complement and coagulation pathways in KD suggests potential therapeutic strategies targeting these systems, which may help regulate inflammation and maintain vascular integrity.

These pathways help regulate the inflammatory response and maintaining vascular integrity, processes that are critically disrupted in KD. The engagement of these systems is subject to a delicate equilibrium and is managed by precise regulatory processes [42]. These systems are essential for an appropriate innate response to injury, curbing hemorrhage and infection, and fostering the healing process [43, 44]. Studies have demonstrated that the triggering of complement and coagulation cascades is a principal pathophysiological mechanism in early-onset severe preeclampsia, as identified through maternal proteomic analysis [45]. Moreover, the complement and coagulation pathways have been linked to chemotherapy responsiveness and overall patient survival rates in soft tissue sarcoma [46].

C6 is part of the membrane attack complex, which plays a crucial role in bacterial lysis [47], while complement component 3 is pivotal for innate immunity and inflammation [48, 49]. C3 is involved in phagocytosis, inflammation, and immunomodulatory processes to destroy infectious microorganisms [50]. Research has shown that complement component 3 was significantly elevated in KD patients [51]. Research has shown that complement component 3 levels are significantly elevated in KD patients and decrease after intravenous immunoglobulin treatment [52]. The main function of α1-Antitrypsin is as an antitrypsin, especially against neutrophil elastase [53]. α1-Antitrypsin, which regulates neutrophil elastase, has been linked to CAL in KD [54]. Our findings suggest that complement components C3, C6, and α1-Antitrypsin could serve as valuable biomarkers for KD, helping to identify patients at risk for CAL and guiding treatment decisions.

In summary, our study provides valuable insights into the molecular mechanisms underlying KD and identifies several potential biomarkers for early diagnosis and disease monitoring. The involvement of the AMPK, PI3K-Akt, and complement and coagulation cascade pathways suggests new therapeutic targets for KD. Clinical trials are needed to evaluate the efficacy of targeting these pathways in improving patient outcomes, particularly in preventing vascular damage and reducing CAL incidence. The identification of biomarkers such as C3, C6, and α1-Antitrypsin could improve early detection of KD, particularly in cases with incomplete or atypical clinical presentations. Further research is needed to validate these biomarkers in larger, multicenter studies and explore their clinical utility in routine diagnostic practice and treatment strategies.

## Conclusion

Significant progress has been made in research on biological markers associated with the diagnosis of KD, but these markers are less specific for the diagnosis of KD. We have identified some KD-related biomarkers through proteomics studies, but these biomarkers still require further multicenter, large-sample clinical studies to be used to diagnose KD. We believe that following extensive validation across various populations, these biomarkers may offer novel perspectives for investigating the etiology and targeted therapy of KD.

## Data Availability

Data will be made available on reasonable reason.

## Abbreviations

KD: Kawasaki disease
C: Control
CAL: Coronary artery lesions
DIA: Data-independent acquisition
LC-MS/MS: Liquid chromatography-tandem mass spectrometry
GO: Gene ontology
BP: Biological processes
CC: Cell composition
MF: Molecular function
KEGG: Kyoto Encyclopedia of Genes and Genomes
FC: Fold Change
